# Oral Antibiotic Preparation Is Associated with Lower Surgical Site Infection After Ileostomy Closure: A Retrospective Cohort Study

**DOI:** 10.3390/jcm15197436

**Published:** 2026-09-24

**Authors:** İsmail Ege Subaşı, Ahmet Topcu, Furkan Saydın

**Affiliations:** Department of General Surgery, Sancaktepe Şehit Prof. Dr. İlhan Varank Training and Research Hospital, University of Health Sciences, Istanbul 34785, Türkiye; dregesubas@gmail.com (İ.E.S.); drahmmettopcu36@gmail.com (A.T.)

**Keywords:** ileostomy closure, stoma reversal, surgical site infection, oral antibiotics, mechanical bowel preparation

## Abstract

**Background/Objectives:** Surgical site infection (SSI) remains a common complication after ileostomy closure. Mechanical bowel preparation (MBP) and oral antibiotic preparation are often bundled together, so their independent contributions to SSI risk are unclear. We evaluated the adjusted associations of oral antibiotic preparation and MBP with SSI after ileostomy closure, a setting in which these two components have rarely been examined separately. **Methods:** We retrospectively reviewed 432 consecutive patients undergoing loop or end ileostomy closure at a single center between January 2022 and December 2025. SSI was defined per Centers for Disease Control and Prevention/National Healthcare Safety Network (CDC/NHSN) criteria. Multivariable logistic regression was used to assess adjusted associations with SSI, with MBP and oral antibiotic preparation included as the exposures of primary interest, adjusting for adjuvant oncologic therapy and closed suction drain use—the covariates associated with SSI on univariate analysis. **Results:** SSI occurred in 35 patients (8.1%). Oral antibiotic preparation was associated with a lower SSI rate (2.9% vs. 11.5% without; absolute risk reduction 8.5 percentage points, unadjusted descriptive number needed to treat 12; *p* = 0.003), while no statistically significant unadjusted association was detected for MBP (*p* = 1.000). After adjustment, oral antibiotic preparation was associated with lower odds of SSI (odds ratio [OR], 0.21; 95% confidence interval [CI], 0.08–0.56; *p* = 0.002), whereas no statistically significant adjusted association was observed for MBP (OR, 1.21; 95% CI, 0.58–2.53; *p* = 0.610); this confidence interval is compatible with both a clinically meaningful benefit and a clinically meaningful harm of MBP, and the study is not powered to exclude either. Adjuvant oncologic therapy (OR 2.53, 95% CI 1.23–5.20) and closed suction drain use (OR 2.33, 95% CI 1.14–4.77) were associated with higher adjusted odds of SSI. The model showed acceptable discrimination (area under the receiver operating characteristic curve [AUC] = 0.745), with no evidence of poor fit on a covariate-pattern-based goodness-of-fit assessment (χ^2^ = 11.37, df = 14, *p* = 0.657). A Firth penalized-likelihood model, used because of the limited number of events in the antibiotic-exposed group, gave materially unchanged estimates (OR 0.23, 95% CI 0.09–0.58). **Conclusions:** Oral antibiotic preparation was associated with lower adjusted odds of SSI after ileostomy closure, whereas the association with MBP remained inconclusive. This observational association should not be interpreted as evidence of causality, because residual confounding—particularly by surgeon-level practice—cannot be excluded. These findings should be considered hypothesis-generating and require confirmation in prospective studies using standardized preparation protocols.

## 1. Introduction

Loop ileostomy is widely constructed to protect high-risk colorectal anastomoses, most commonly following low anterior resection, and mitigates the clinical consequences of anastomotic leakage. Closure of this protective stoma, however, remains a substantial source of morbidity: reported SSI rates after ileostomy closure range widely across studies (approximately 2% to 41%) [1]. Most work aimed at reducing this risk has focused on wound closure technique—purse-string closure reduces SSI compared with conventional linear closure in randomized trials and a Cochrane review [2,3], and circular closure and adjuncts such as negative-pressure wound therapy have shown further benefit [4,5,6,7,8].

The role of preoperative bowel preparation is far less settled. In primary colorectal resection, combined MBP and oral antibiotic preparation reduce SSI, anastomotic leak, and ileus [9,10], but several analyses of the literature attribute most of this benefit to the oral antibiotic component rather than to mechanical clearance itself, since MBP alone has not consistently reduced SSI in randomized trials [9,10]. Almost all of this evidence comes from operations that create a fresh anastomosis; ileostomy closure occurs in a different setting, centered on skin and subcutaneous tissue around a stoma already colonized by enteric and cutaneous flora. Unlike the colon, which continuously accumulates formed stool, the diverted small bowel in an ileostomy retains little solid content even without mechanical preparation, so the mechanistic rationale for MBP is inherently weaker in this setting than in colorectal resection. Pediatric randomized trials have found no benefit of MBP before elective bowel resection or ostomy closure [11,12], and an enhanced-recovery pathway omitting MBP before stoma reversal was feasible and safe [13]. A recent single-center series empirically combined oral antimicrobials with technique modifications for stoma closure and reported a low SSI rate, but was underpowered—two events among 92 patients—to identify independent risk factors [14]. Whether oral antibiotic preparation, independent of mechanical preparation, is associated with SSI after ileostomy closure remains insufficiently examined.

We therefore examined whether MBP and oral antibiotic preparation were independently associated with SSI after ileostomy closure.

## 2. Materials and Methods

### 2.1. Study Design, Population, and Eligibility

This retrospective cohort study included patients undergoing ileostomy closure between January 2022 and December 2025, comprising 432 consecutive patients who underwent loop or end ileostomy closure at Sancaktepe Şehit Prof. Dr. İlhan Varank Training and Research Hospital, University of Health Sciences, Istanbul, Türkiye. Ethics approval was obtained on 12 November 2025 (Sancaktepe Şehit Prof. Dr. İlhan Varank Training and Research Hospital Scientific Research Ethics Committee, Decision No.: 2025/416) before initiation of any study-specific chart review or data extraction. Data extraction commenced on 17 November 2025 and was finalized on 20 February 2026, after 30-day follow-up had been completed for all eligible patients, including those operated on in December 2025; patient data were anonymized prior to analysis. This report follows the STROBE statement for cohort studies [15]; the patient-flow diagram is presented in Figure 1, and the completed STROBE checklist is provided as Supplementary Table S1.

Patients were identified from the hospital’s electronic surgical scheduling system for the study period. Of 487 patients assessed for eligibility, 55 were excluded: age under 18 years (*n* = 3); permanent stoma conversion rather than closure (*n* = 18); fewer than 30 days of documented postoperative follow-up (*n* = 14); and missing records, in particular missing SSI outcome data or missing data for core covariates (*n* = 20). During screening, additional technical exclusion criteria were also applied within these categories: repeat closure attempts, care transferred from another center for follow-up only, intraoperative conversion to a new stoma, inability to perform primary closure because of active intra-abdominal sepsis, damage-control procedures, and a major additional abdominal resection in the same session. Individual per-category counts for these six technical criteria were not separately tabulated in the screening log and are therefore not reported; the reported total of 55 exclusions in Figure 1 is unaffected. No patient in the resulting cohort of 432 had missing data for the primary outcome or for any covariate used in the univariate or multivariable analyses; all 432 records were analyzed without imputation.

There was no standardized institutional protocol for either mechanical bowel preparation or oral antibiotic preparation at any point during the study period. The decision to administer either preparation, and the choice of regimen, were made at the discretion of the attending surgeon. When performed, MBP consisted of a self-administered oral osmotic or stimulant laxative regimen the day before surgery; oral antibiotic preparation, when given, followed no single fixed regimen, and the specific agents, doses, and timing relative to surgery were not captured in a standardized, analyzable field (see Limitations). MBP and oral antibiotic preparation status were determined from the electronic medical record; compliance with self-administered MBP regimens taken at home could not be independently verified. Because preparation was determined by the attending surgeon rather than a standardized protocol, residual confounding by surgeon-level practice and case selection cannot be excluded. Because surgeon identity was not captured as a structured variable in the analysis dataset, surgeon-level adjustment or clustering could not be performed; accordingly, the estimated association between oral antibiotic preparation and SSI should be interpreted as an adjusted observational association rather than a causal treatment effect. A further potential source of bias is that SSI ascertainment relied on presentation to, or documentation within, the treating institution; superficial infections managed entirely elsewhere after discharge could have been missed, which would tend to underestimate true incidence, particularly for milder cases.

### 2.2. Outcome Variable and Definitions

The primary outcome was SSI occurring within 30 postoperative days, classified per CDC/NHSN criteria as superficial incisional, deep incisional, or organ/space SSI. Secondary outcomes were SSI-related reintervention, length of hospital stay, 30-day readmission, and 30-day mortality. SSI-related reintervention was recorded in the source dataset as a binary field (yes/no) without a linked date, procedure type, or explicit relationship to hospital discharge; we therefore describe it descriptively in Results and avoid assuming it represents a formally dated, timed “reoperation” (i.e., a confirmed return to the operating room) as opposed to a bedside wound intervention such as debridement or drainage—see Limitations.

### 2.3. Variables

Recorded variables included demographic characteristics (age, sex, height, weight, BMI); comorbidities (smoking, nutritional status, ASA class, diabetes, immunosuppression); initial stoma indication and type; ostomy duration; oncologic treatment history; preoperative preparation (MBP, oral antibiotic preparation, prophylactic intravenous antibiotics); and postoperative variables (SSI type, day of diagnosis, wound culture, isolated microorganism, SSI treatment). A further set of intraoperative variables—bowel segment opened, perfusion-assessment method, fascial defect size, subcutaneous fat thickness, surgical approach, anastomotic technique, wound closure method, drain use, estimated blood loss, and intraoperative complications—was also recorded; these are summarized in Table 1 and Section 2.4, and the complete set of intraoperative variables and their univariate association with SSI is provided in Supplementary Table S6. Suture material was not captured as a structured, analyzable variable and is discussed qualitatively in Section 2.4/Limitations. Immunosuppression was recorded but was absent in the entire cohort (0/432) and was therefore not included in comparative analyses. Steroid exposure was not captured as a separate structured variable and could not be analyzed. Inflammatory bowel disease (IBD) was identified from the recorded initial stoma indication field (36/432 patients, 8.3% of the cohort) and was evaluated in an additional sensitivity analysis (Section 2.5, Supplementary Table S2(f)) because of its potential relationship with both perioperative management and infectious outcomes.

Nutritional status was categorized by the attending surgical team at preoperative assessment based on clinical judgement, rather than a validated screening instrument such as NRS-2002 or MUST (weight loss history, oral intake, and serum albumin where available); “poor nutritional status” in Table 1 and Table 2 merges the moderate-risk and malnutrition categories (144 patients, 33.3% of the cohort).

### 2.4. Wound Closure Technique

Two principal wound closure techniques were used: purse-string closure (46.8%) and primary (linear) closure (47.0%), with a small subset (6.3%) left open to heal by secondary intention; no statistically significant overall association was observed between wound-closure technique and SSI across the three categories (Table 2; Fisher–Freeman–Halton *p* = 0.784). The general operative steps included a circumferential mucocutaneous incision around the stoma, mobilization of the efferent and afferent limbs from the abdominal wall, resection or hand-sewn/stapled closure of the stoma-bearing bowel segment, restoration of bowel continuity, and skin closure by the purse-string, primary, or open technique; the timing of stoma-aperture closure or compression relative to the start of the procedure was not separately standardized or recorded. Anastomotic technique (stapled/circular, hand-sewn, or combined) and perfusion-assessment method showed no significant association with SSI (*p* = 0.066 and *p* = 0.654, respectively), whereas closed suction drain use was associated with SSI (*p* = 0.021) and was retained as a covariate in the multivariable model (Section 2.5, Table 3). In the microbiology field underlying Table 4 and Supplementary Table S5, each positive culture record contains either a single organism or an explicit “polymicrobial” designation; anaerobic culture was not requested as a routine, standing order for wound specimens at this institution, which would be expected to underestimate anaerobic and polymicrobial involvement. Suture material and the specific technique for each closure type were not standardized across the cohort, which we acknowledge as a limitation. Representative postoperative images of purse-string and primary closure are shown in Figure 2.

### 2.5. Statistical Analysis

Normality of continuous variables was assessed with the Shapiro-Wilk test; the independent-samples *t*-test or Mann–Whitney U test was used accordingly. Categorical variables were compared with the Yates-corrected chi-square test for 2 × 2 tables in which all expected cell counts were ≥5, or Fisher’s exact test for 2 × 2 tables with any expected cell count <5; the three-category comparison of wound closure technique with SSI, which had an expected cell count below five in the left-open group, was assessed with the Fisher–Freeman–Halton exact test; multi-category comparisons (e.g., ASA class, nutritional status) used the uncorrected chi-square test across all levels. Univariate comparisons were not adjusted for multiple testing and should be regarded as exploratory.

The primary analysis was a multivariable logistic regression model in which MBP and oral antibiotic preparation—the exposures of primary interest—were entered together with adjuvant oncologic therapy and closed suction drain use, the covariates associated with SSI on univariate analysis at *p* < 0.05. As a sensitivity analysis, we fit a backward-elimination model (removal threshold *p* > 0.05) using all variables significant at *p* < 0.05 on univariate analysis (oral antibiotic preparation, adjuvant oncologic therapy, closed suction drain use), without forcing in MBP. Operative duration was also nominally significant on univariate analysis (71 vs. 79 min, *p* = 0.023); rather than excluding it a priori, we fit an additional sensitivity model forcing in operative duration alongside MBP, oral antibiotic preparation, adjuvant oncologic therapy, and closed suction drain use. We also fit a further sensitivity model forcing in inflammatory bowel disease (IBD) as the initial stoma indication, given its imbalanced distribution across preparation groups. Because of the limited number of events in the oral-antibiotic-exposed group (five SSI events among 170 patients), we additionally fit the primary model using Firth penalized-likelihood logistic regression as a robustness check for sparse-data bias, and—to address whether diabetes and ASA class might still explain part of the association despite not reaching significance on the corrected univariate analysis—a model forcing in diabetes and ASA class (III–IV vs. I–II) alongside MBP and oral antibiotic preparation.

Model performance was assessed using the area under the receiver operating characteristic curve (AUC), McFadden’s pseudo-R^2^, and the events-per-variable (EPV) ratio, reported for both the primary and sensitivity models given the limited number of outcome events; ROC curves are compared in Figure 3. The primary model is reported as primary because it contains the pre-specified exposures of interest (MBP, oral antibiotic preparation) together with the covariates significant on univariate analysis, not because of superior discrimination; no formal comparison of the primary and sensitivity AUCs is made. For the primary model, we additionally assessed multicollinearity using variance inflation factors (all < 1.1), goodness-of-fit using the Hosmer–Lemeshow test [16], computed across distinct covariate-pattern groups (rather than by decile, which is not meaningful for a model with a small number of unique predicted values) using a custom implementation of the Pearson-type goodness-of-fit statistic, and calibration by plotting observed versus predicted risk by covariate-pattern group (Figure 4). A *p*-value < 0.05 was considered statistically significant throughout. Analyses were performed in Python 3.12 (pandas 3.0.2, scipy 1.17.1, statsmodels 0.15.0, scikit-learn 1.8.0).

As part of the present revision, the complete statistical analysis was re-run from the locked, de-identified patient-level analysis dataset (*n* = 432). All reported univariate comparisons, multivariable models, sensitivity analyses, interaction analyses, ROC estimates, and calibration analyses were regenerated directly from this dataset; Table 1 and Table 4 were regenerated using the same code and dataset as all other tables and figures. No manuscript-derived or manually entered result values were used as inputs to the analysis. The complete executable analysis code is provided as Supplementary File S1 to facilitate reproducibility.

**Table 3:** Multivariable logistic regression models for SSI.

**Primary Model**			
**Variable**	**OR**	**95% CI**	***p***
Mechanical bowel preparation (ref: not performed)	1.21	0.58–2.53	0.610
Oral antibiotic preparation (ref: not performed)	0.21	0.08–0.56	0.002
Adjuvant oncologic therapy (ref: no)	2.53	1.23–5.20	0.011
Closed suction drain used (ref: not placed)	2.33	1.14–4.77	0.021
**Sensitivity model (backward elimination, MBP not forced in)**			
**Variable**	**OR**	**95% CI**	***p***
Oral antibiotic preparation	0.22	0.08–0.58	0.002
Adjuvant oncologic therapy	2.50	1.22–5.14	0.012
Closed suction drain used	2.35	1.15–4.80	0.019
**Diabetes/ASA-adjusted model**			
**Variable**	**OR**	**95% CI**	***p***
Mechanical bowel preparation	1.21	0.59–2.49	0.604
Oral antibiotic preparation	0.22	0.08–0.60	0.003
Diabetes	2.37	0.90–6.28	0.083
ASA III–IV (ref: I–II)	0.81	0.31–2.10	0.667
**Operative-duration-adjusted sensitivity model**			
**Variable**	**OR**	**95% CI**	***p***
Mechanical bowel preparation	1.223	0.58–2.57	0.595
Oral antibiotic preparation	0.218	0.080–0.588	0.003
Operative duration (per minute)	0.976	0.957–0.995	0.014
Adjuvant oncologic therapy	2.698	1.30–5.62	0.008
Closed suction drain used	2.587	1.24–5.39	0.011
**IBD-adjusted sensitivity model**			
**Variable**	**OR**	**95% CI**	***p***
Mechanical bowel preparation	1.217	0.57–2.61	0.613
Oral antibiotic preparation	0.208	0.077–0.561	0.002
IBD (initial stoma indication)	0.931	0.197–4.406	0.928
**Firth penalized-likelihood model (primary model)**			
**Variable**	**OR**	**95% CI**	***p***
Mechanical bowel preparation	1.20	0.58–2.45	0.627
Oral antibiotic preparation	0.23	0.09–0.58	0.002
Adjuvant oncologic therapy	2.49	1.23–5.01	0.011
Closed suction drain used	2.29	1.14–4.61	0.020
**Model summary statistics**			
**Metric**	**Primary model (4 covariates)**		**Sensitivity model (3 covariates)**
Events per variable (EPV)	8.75		11.67
AUC	0.745		0.742
Pseudo R^2^ (McFadden)	0.099		0.098
Hosmer–Lemeshow χ^2^(14), *p*	11.37, df = 14, *p* = 0.657		—
Max variance inflation factor	1.03		—

Reference category for every binary covariate is “No/Not performed.” BMI, poor nutritional status, and smoking were candidate covariates in the original analysis plan but were not significant on the corrected univariate analysis (Table 2) and were not entered into the primary model.

**Table 4:** Clinical course and microbiological profile of patients who developed SSI (*n* = 35).

Parameter	Result	Note
SSI type—Superficial incisional	26 (74.3%)	
SSI type—Deep incisional	8 (22.9%)	
SSI type—Organ/space	1 (2.9%)	
Day of SSI diagnosis, median (IQR)	11.0 (7.0–13.0)	Postoperative day
Wound culture obtained	25 (71.4%)	10/35 not cultured
Culture-negative (no growth)	5 (14.3% of 35)	
Polymicrobial growth	2 (5.7% of 35)	
*Enterococcus faecalis*	7 (20.0% of 35)	Most frequent single isolate
*Escherichia coli*	6 (17.1% of 35)	
*Staphylococcus aureus*	3 (8.6% of 35)	
*Klebsiella pneumoniae*	2 (5.7% of 35)	
SSI-related reintervention *	10 (28.6%)	Not applicable in SSI(−) group by definition
30-day readmission	3 (8.6%)	SSI(−): 23/397 (5.8%), *p* = 0.435 (Fisher)
Length of hospital stay (SSI+), median (IQR)	9.0 (8.0–10.0) days	SSI(−): 6.0 (4.0–7.0) days, MWU *p* < 0.001
30-day mortality (whole cohort)	1/432 (0.2%)	Occurred in SSI(−) group

Anaerobic culture was not performed as a routine, standing order for wound specimens at this institution (see Section 2.3). Each positive culture record contains either a single organism or an explicit “polymicrobial” designation. * The source field records only a binary yes/no reintervention flag with no linked date or procedure type; see Section 2.2 and Limitations. Of the 10 patients with a reintervention record, six had a day of SSI diagnosis that exceeded their length of stay, but the exact timing and type of the reintervention itself is not recorded, so it cannot be assumed to have occurred predominantly during the index admission.

## 3. Results

The median age of the 432 patients was 59.5 years (IQR, 51.0–68.2), and 61.6% were male. Loop ileostomy was present in 92.4% and end ileostomy in 7.6%; the most common initial indication was rectal cancer (33.1%). Cohort characteristics are summarized in Table 1.

The overall SSI incidence was 8.1% (35/432) (Table 2). On univariate analysis, higher BMI showed a numerical trend that did not reach significance (28.6 ± 4.5 vs. 27.4 ± 3.7 kg/m^2^, *p* = 0.088), as did age (median 63 vs. 59 years, *p* = 0.458), ASA class III–IV (*p* = 0.596), diabetes (*p* = 0.093), poor nutritional status (*p* = 0.493), and smoking (*p* = 0.734)—none of which reached conventional significance in the corrected analysis. Adjuvant oncologic therapy (57.1% vs. 35.3%, *p* = 0.017) and closed suction drain use (54.3% vs. 33.5%, *p* = 0.021) were significantly more common among patients who developed SSI. Female sex, neoadjuvant therapy, MBP, prophylactic intravenous antibiotics, and wound closure technique showed no association with SSI. In contrast, oral antibiotic preparation was associated with a significantly lower SSI rate (2.9% vs. 11.5%; *p* = 0.003; absolute risk reduction 8.5 percentage points; unadjusted descriptive number needed to treat 12)—the only preparation modality significantly associated with SSI in either direction.

On multivariable analysis, the primary model—which entered MBP and oral antibiotic preparation together with adjuvant oncologic therapy and closed suction drain use—showed oral antibiotic preparation to be associated with lower adjusted odds of SSI (OR 0.21, 95% CI 0.08–0.56; *p* = 0.002), whereas no statistically significant adjusted association was observed for MBP (OR 1.21, 95% CI 0.58–2.53; *p* = 0.610) (Table 3).

The sensitivity model, obtained by backward elimination without forcing in MBP or oral antibiotic preparation, retained oral antibiotic preparation, adjuvant oncologic therapy, and drain use, with similar adjusted associations across model specifications (AUC 0.745 vs. 0.742). A model additionally forcing in diabetes and ASA class (III–IV vs. I–II) alongside MBP and oral antibiotic preparation gave a materially unchanged estimate for oral antibiotic preparation (OR 0.22, 95% CI 0.08–0.60, *p* = 0.003), while diabetes (OR 2.37, 95% CI 0.90–6.28, *p* = 0.083) and ASA III–IV (OR 0.81, 95% CI 0.31–2.10, *p* = 0.667) were not statistically significant after adjustment. A Firth penalized-likelihood logistic regression, fitted because of the limited number of events in the antibiotic-exposed group (5 SSI events among 170 patients), gave materially unchanged estimates for the primary model (oral antibiotic preparation OR 0.23, 95% CI 0.09–0.58, *p* = 0.002); full model output for all four models is provided in Supplementary Table S2. ROC curves for the primary and sensitivity models are compared in Figure 3.

In an operative-duration-adjusted sensitivity model forcing in operative duration alongside MBP, oral antibiotic preparation, adjuvant oncologic therapy, and closed suction drain use, oral antibiotic preparation remained associated with lower adjusted odds of SSI (OR 0.218, 95% CI 0.080–0.588, *p* = 0.003), and operative duration was associated with lower adjusted odds of SSI (OR 0.976 per minute, 95% CI 0.957–0.995, *p* = 0.014); adjustment for operative duration did not materially alter the estimated association with oral antibiotic preparation. In a separate sensitivity model forcing in IBD as the initial stoma indication alongside MBP and oral antibiotic preparation, oral antibiotic preparation remained associated with lower adjusted odds of SSI (OR 0.208, 95% CI 0.077–0.561, *p* = 0.002), while no statistically significant adjusted association was observed for IBD (OR 0.931, 95% CI 0.197–4.406, *p* = 0.928).

The primary model showed no evidence of problematic multicollinearity (all variance inflation factors < 1.1); the Hosmer–Lemeshow test, computed across the 16 distinct covariate-pattern groups produced by the four binary predictors (rather than by decile, which is not meaningful for a model with only 16 unique predicted values), showed no evidence of poor fit (χ^2^ = 11.37, df = 14, *p* = 0.657), and the calibration plot (Figure 4) showed reasonable agreement between predicted and observed risk across these 16 covariate-pattern groups, each with its own group size and observed/expected SSI count.

Full model output, including the backward-elimination sequence, the diabetes/ASA-adjusted model, and the Firth penalized-likelihood model, the completed STROBE checklist, and ROC coordinate data, are provided in Supplementary Materials (Tables S1–S3).

The clinical course of the 35 SSI cases is summarized in Table 4: 74.3% were superficial incisional, 22.9% deep incisional, and 2.9% organ/space SSI. The median day of diagnosis was postoperative day 11 (IQR 7–13). A wound culture was obtained in 25/35 (71.4%) of SSI cases; among these, five (14.3% of all SSI cases) were culture-negative and two (5.7%) were polymicrobial. Among single-organism cultures, the most frequent isolate was *Enterococcus faecalis* (seven), followed by *Escherichia coli* (six), *Staphylococcus aureus* (three), and *Klebsiella pneumoniae* (two). SSI-related reintervention was recorded in 10 of 35 patients with SSI (28.6%). Six of these patients had an SSI diagnosis date later than their recorded length of stay. However, because the date and type of reintervention were not captured, it was not possible to determine whether these interventions were performed during the index admission, after readmission, or in an outpatient setting. Median length of stay was longer in the SSI(+) group (9 [IQR 8–10] days) than in the SSI(−) group (6 [IQR 4–7] days; Mann–Whitney U *p* < 0.001); because median length of stay (9 days) was shorter than the median day of SSI diagnosis (11 days), diagnosis occurred after discharge (day of diagnosis exceeding length of stay) in 18/35 (51.4%) of all SSI cases. Thirty-day readmission did not differ significantly by SSI status (8.6% vs. 5.8%, Fisher’s exact *p* = 0.435). One death occurred in the whole cohort within 30 days (1/432, 0.2%), in the SSI(−) group.

A cross-tabulation of the two preparation modalities (Table 5) shows that oral antibiotic preparation was associated with a lower SSI incidence within both the MBP-performed stratum (2.5% vs. 12.8%) and the MBP-not-performed stratum (3.8% vs. 9.9%), supporting an independent protective association not explained by co-administration of mechanical bowel preparation.

A formal MBP × oral antibiotic interaction term was tested. No statistically significant interaction was detected between MBP and oral antibiotic preparation (interaction OR = 0.49, *p* = 0.480), indicating no formal evidence of effect-measure modification between the two preparation modalities; the pattern in Table 5 should therefore be interpreted as two main effects rather than a confirmed interaction.

**Table 5:** SSI incidence by combined mechanical bowel preparation (MBP) and oral antibiotic (Oral) preparation status.

Group	N	SSI Events	SSI Incidence	95% CI (Wilson)
MBP+/Oral+	118	3	2.5%	0.9–7.2%
MBP+/Oral−	141	18	12.8%	8.2–19.3%
MBP−/Oral+	52	2	3.8%	1.1–13.0%
MBP−/Oral−	121	12	9.9%	5.8–16.5%

Oral antibiotic preparation is associated with a lower SSI incidence within both the MBP-performed and MBP-not-performed strata.

## 4. Discussion

In this cohort of 432 patients undergoing ileostomy closure, oral antibiotic preparation was associated with substantially lower adjusted odds of SSI, whereas no statistically significant adjusted association was observed for MBP (adjusted OR 0.21, 95% CI 0.08–0.56 vs. OR 1.21, 95% CI 0.58–2.53); this confidence interval for MBP is compatible with both a clinically meaningful benefit and a clinically meaningful harm, and the study is not powered to exclude a clinically relevant effect of MBP. As detailed in the Limitations, this association is observational and derived from surgeon-directed rather than randomized allocation of preparation; it should therefore be interpreted as hypothesis-generating rather than as evidence of a causal treatment effect. The overall SSI incidence in this cohort was 8.1%, toward the lower end of the range reported in the literature (2–41%) [1]. Wound closure technique is a major recognized driver of this variability—purse-string closure has repeatedly outperformed linear closure in randomized evidence [2,3,5]—though in the present cohort the corrected SSI incidence by closure technique was 8.4% for purse-string closure, 7.4% for primary closure, and 11.1% for wounds left open (Table 2); this is not a gradient favouring purse-string closure and the difference was not statistically significant (Fisher–Freeman–Halton *p* = 0.784). We do not consider this cohort adequately powered to test closure-technique effects, and present these findings descriptively rather than as evidence for or against purse-string closure’s benefit reported elsewhere.

These findings are consistent with evidence from primary colorectal resection suggesting that the oral antibiotic component, rather than mechanical clearance itself, accounts for much of the benefit historically attributed to combined bowel preparation: large trials and meta-analyses show that MBP plus oral antibiotics reduces SSI, anastomotic leak, and ileus [9,10], but several analyses of that evidence attribute this benefit specifically to the antibiotic component, since MBP alone has not reliably reduced SSI in randomized comparisons [9,10]. The independent contribution of oral antibiotic preparation, separate from MBP, remains insufficiently characterized specifically in ileostomy closure; a recent single-center bundle-based series that empirically combined oral antimicrobials with technique modifications in stoma closure reported a low SSI rate but was underpowered—two events among 92 patients—to identify independent risk factors, and did not separately estimate the association of oral antibiotic preparation with SSI [14].

Unlike the colon, which continuously accumulates formed stool, the diverted small bowel in an ileostomy retains little solid content even without mechanical preparation; residual stool is more characteristic of elective colorectal resection and colostomy closure, where the perioperative infection risk is plausibly driven more by fecal bulk. This distinction provides a plausible biological rationale for why oral antibiotic preparation may be associated with SSI risk independently of mechanical evacuation in the ileostomy-closure setting studied here. However, the present observational data cannot establish the mechanism underlying this association, and our organism profile (mixed enteric and cutaneous flora, including culture-negative and polymicrobial results; Table 4, Supplementary Table S5) is more consistent with a small-bowel/stoma source than with the more heavily gram-negative and anaerobic flora typically reported after colonic surgery. Our finding is compatible with pediatric randomized evidence showing no benefit of MBP alone [11,12] and with a trial showing that an enhanced-recovery pathway omitting MBP before stoma reversal was feasible and safe [13], since neither of those studies isolated the antibiotic component as we did here. If confirmed prospectively, these findings question the benefit of routine MBP alone and support prospective evaluation of oral antibiotic preparation in ileostomy closure.

Beyond the exposures examined here, other cohorts and reviews have described a broader set of risk factors for SSI after stoma reversal and colorectal surgery, including a systematic review and meta-analysis of SSI incidence and risk factors after stoma reversal [17], a predictive nomogram incorporating multiple perioperative variables specifically for SSI after loop ileostomy closure [18], obesity and higher BMI [19,20], and general risk-factor analyses in colorectal surgery [21,22]; the American Society of Colon and Rectal Surgeons has summarized preventive strategies drawn from this literature in its clinical practice guidelines [23], and a comparably multifactorial risk profile has been described for anastomotic leakage after stoma closure [24]. We did not find female sex or neoadjuvant therapy to be associated with SSI in this cohort, which is broadly consistent with larger meta-analyses and surveillance data [25,26,27]; a cohort of this size and event count (35 SSI events) has limited power to detect modest effects for any single variable, and these null findings should not be read as evidence against their relevance elsewhere.

Isolated organisms (*Enterococcus faecalis*, *Escherichia coli*, *Staphylococcus aureus*, *Klebsiella pneumoniae*), together with the culture-negative and polymicrobial results now reported in Table 4, reflected a mixed enteric and cutaneous flora, as expected for the stoma site. SSI-related reintervention was recorded in 28.6% of SSI cases, illustrating its clinical burden among affected patients. Length of stay was longer in patients with SSI, whereas 30-day readmission did not differ significantly between groups. We interpret the readmission finding cautiously rather than as evidence that SSI carries no burden beyond the index admission: the median day of SSI diagnosis (postoperative day 11) exceeded the median length of stay (9 days) in the SSI(+) group, and diagnosis occurred after discharge in just over half of SSI cases (18/35, 51.4%), suggesting many SSIs were managed as outpatients for predominantly superficial infections (74.3% of cases) without triggering a formal 30-day readmission—a pattern that warrants confirmation with individual-level, post-discharge surveillance data including reintervention dates and procedure type, since ascertainment bias could also contribute to an underestimate of SSI-attributable morbidity.

This study has several limitations. The single-center, retrospective design cannot exclude unmeasured confounding, including surgeon experience, volume, and case complexity, none of which were captured as structured variables. Surgeon identity was not available as a structured variable; therefore, surgeon-level clustering could not be incorporated into the analysis. Because preparation and closure technique were chosen at surgeon discretion rather than randomized, residual confounding by surgeon-level practice and case selection cannot be excluded; women were somewhat more frequent in the MBP-performed group (43.2% vs. 31.2%, *p* = 0.016; Supplementary Table S4), which we cannot fully rule out as a residual confounder. This limitation is particularly relevant because both MBP and oral antibiotic preparation were determined at the discretion of the attending surgeon; the observed association may therefore partly reflect correlated surgeon-level practices, including patient selection, perioperative management, wound management, or other unmeasured practice patterns, rather than the isolated effect of either preparation component. MBP and oral antibiotic preparation status were determined from the electronic medical record and could not be independently verified for compliance; non-differential misclassification of either binary exposure may attenuate associations toward the null under conventional assumptions; however, the magnitude and direction of bias cannot be established with certainty in this observational dataset. Differential documentation quality by surgeon or calendar year could bias the estimate in either direction, and we cannot exclude this. The specific antibiotic agents, doses, and timing were not captured and could not be analyzed, limiting our ability to identify which regimen underlies the observed association—a priority for future protocol-level data collection. Adjuvant oncologic therapy was recorded as a binary yes/no field without an administration date; whether treatment always preceded ileostomy closure, and the interval between adjuvant treatment and closure, could not be confirmed as a structured variable. Because the timing of adjuvant oncologic therapy relative to ileostomy closure was not available as a structured variable, this covariate should be interpreted as an associated clinical characteristic rather than a temporally established preoperative risk factor. Nutritional status was categorized by clinical judgement rather than a validated instrument (e.g., NRS-2002 or MUST), and “poor nutritional status” merges the moderate-risk and malnutrition categories (144 patients). Established modifiable factors such as wound protector use [28,29] and subcutaneous wound irrigation [30,31,32] were not recorded and could not be adjusted for. With only 35 SSI events, the EPV ratio was limited (8.75 in the primary model, 11.67 in the sensitivity model); a Firth penalized-likelihood sensitivity analysis yielded a materially similar estimate, suggesting that the observed association was not solely attributable to small-sample maximum-likelihood bias; the confidence interval nonetheless remains wide (0.08–0.56), and the finding should be treated as hypothesis-generating rather than definitive. Model discrimination (AUC = 0.745) indicates that unmeasured factors likely contribute to residual risk. SSI-related reintervention was recorded only as a binary field without a linked date or procedure type: six of the 10 recorded reinterventions occurred in patients whose SSI diagnosis date exceeded their length of stay, but the exact timing and type of each reintervention itself is not recorded, so we could not determine the proportion occurring before versus after discharge, or distinguish a formal return to the operating room from a bedside wound intervention; future data collection should capture this explicitly. Finally, SSI ascertainment depended on presentation to or documentation within the treating institution, which may underestimate true incidence for milder, externally managed infections, and single-center data may not generalize to centers with different bowel preparation, antibiotic, or wound-management protocols.

## 5. Conclusions

In this retrospective cohort, oral antibiotic preparation was associated with lower adjusted odds of SSI after ileostomy closure, whereas the association between MBP and SSI remained inconclusive; the confidence interval was compatible with clinically relevant effects in either direction, and this study is not powered to exclude either. This observational association should not be interpreted as evidence of causality, because residual confounding—particularly by surgeon-level practice, given that both preparations were administered at surgeon discretion—cannot be excluded. These findings suggest that the antimicrobial and mechanical components of bowel preparation should not necessarily be considered equivalent in this setting, and should be regarded as hypothesis-generating. Given the retrospective design, limited number of SSI events, lack of standardized antibiotic regimens, and inability to adjust for surgeon-level clustering, prospective multicenter studies using standardized preparation protocols are required before changes to routine practice can be recommended.

## Figures and Tables

**Figure 1:**
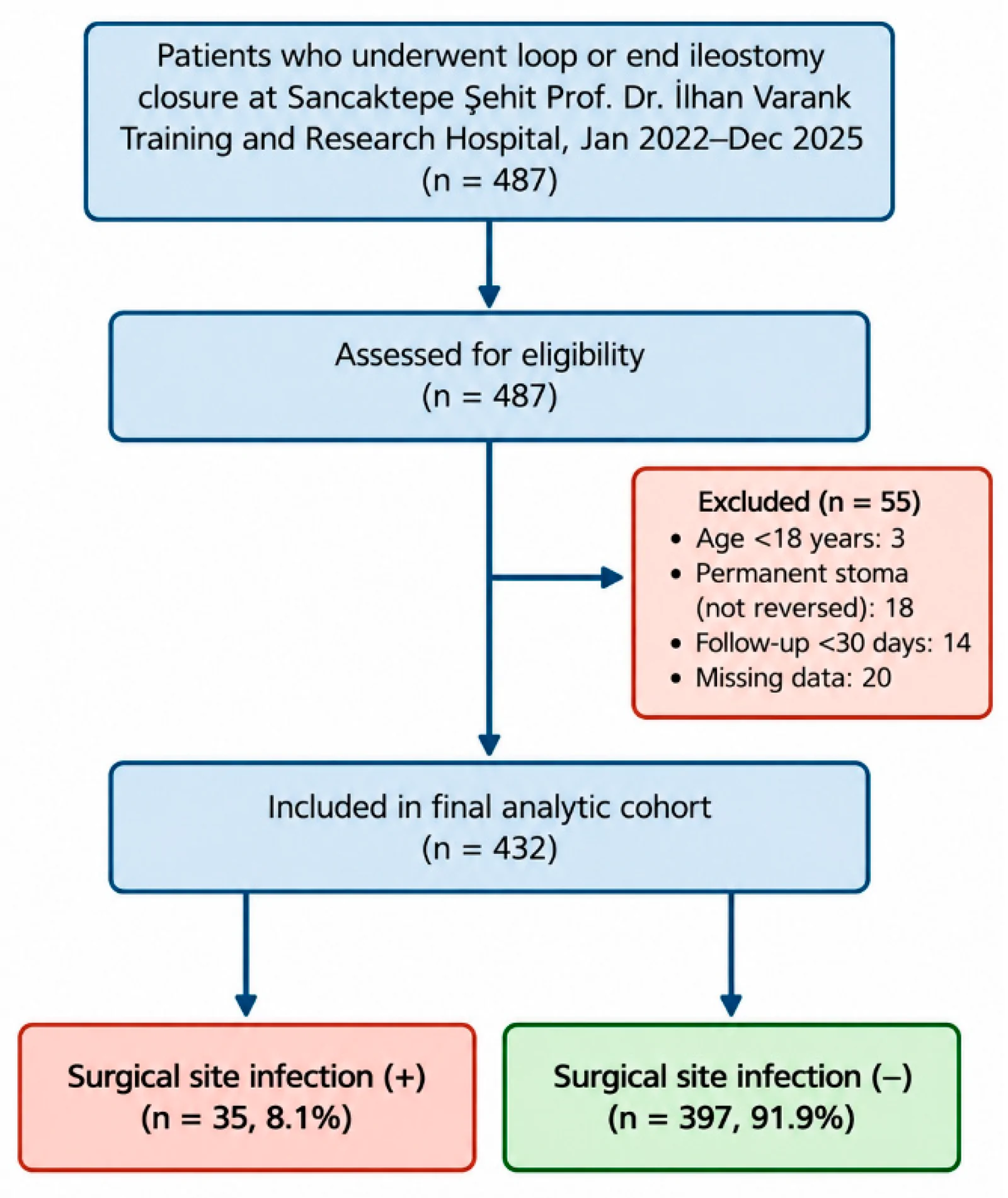
STROBE flow diagram of patient selection.

**Figure 2:**
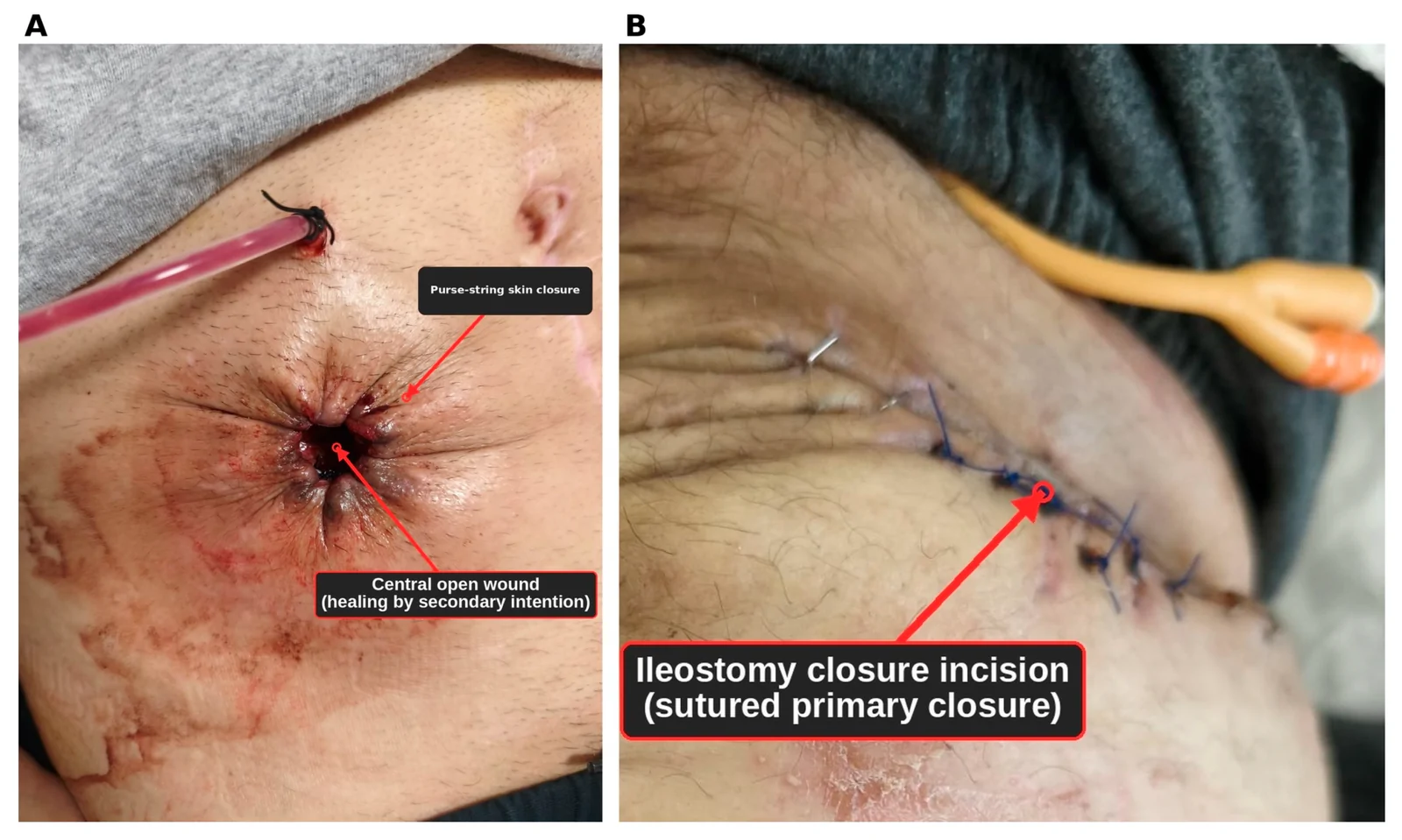
Representative postoperative appearance following (**A**) purse-string skin closure, demonstrating the characteristic central wound opening left to heal by secondary intention, and (**B**) conventional primary linear skin closure after ileostomy reversal. Written informed consent for publication of these clinical images was obtained from the patients.

**Figure 3:**
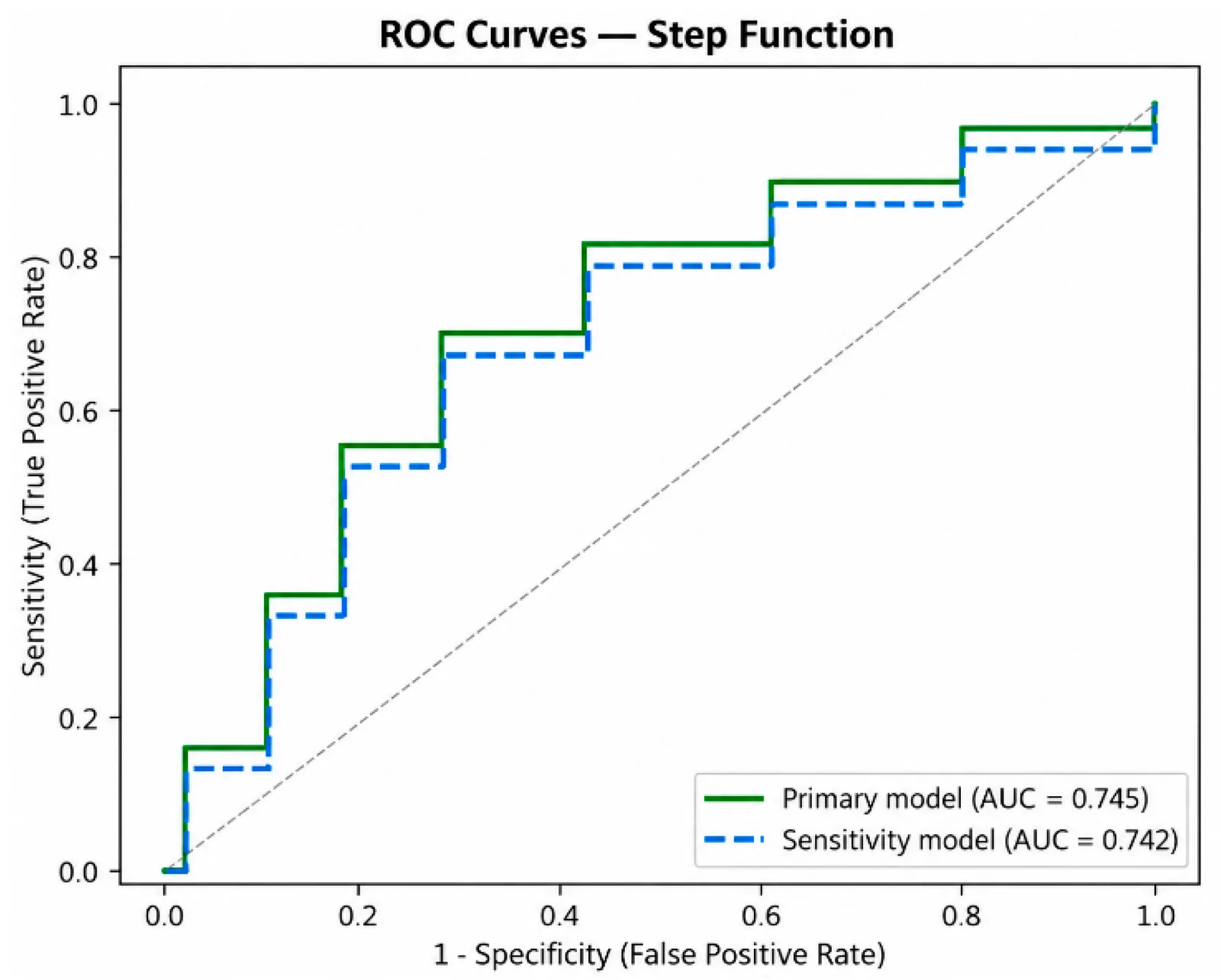
Receiver operating characteristic (ROC) curves, redrawn as step functions, for the primary model (MBP, oral antibiotic preparation, adjuvant oncologic therapy, closed suction drain; AUC = 0.745) and the sensitivity model (oral antibiotic preparation, adjuvant oncologic therapy, closed suction drain; AUC = 0.742).

**Figure 4:**
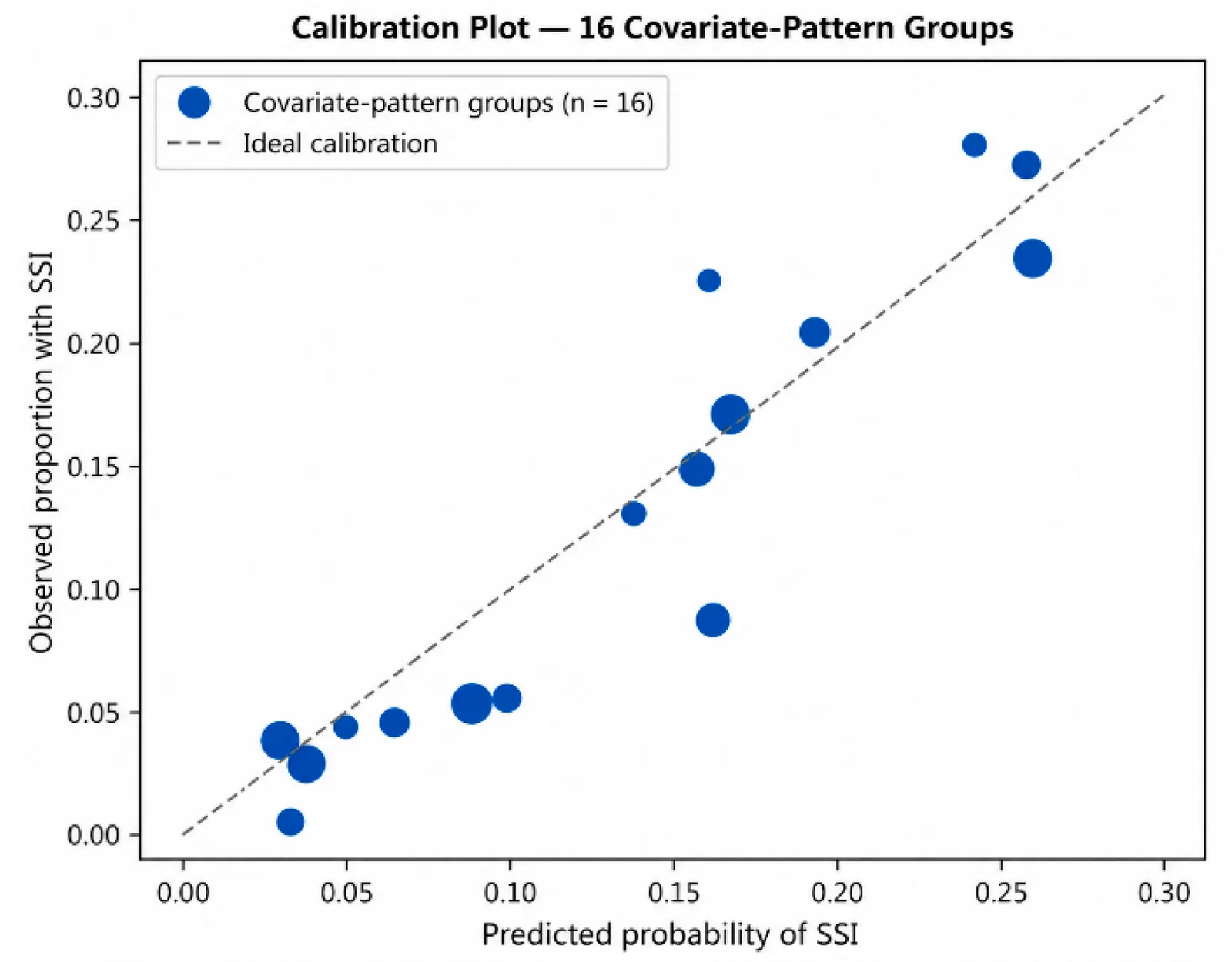
Calibration plot for the primary model, showing observed versus predicted probability of SSI across the 16 distinct covariate-pattern risk groups produced by the model. Circle size represents the number of patients within each covariate-pattern group; the dashed diagonal represents ideal calibration. Points close to the diagonal indicate good agreement between predicted and observed risk.

**Table 1:** Demographic and clinical characteristics of the entire cohort (*n* = 432).

Variable	Entire Cohort (*n* = 432)
Age (years), median (IQR)	59.5 (51.0–68.2)
BMI (kg/m^2^), mean ± SD	27.5 ± 3.7
Ostomy duration (days), median (IQR)	173.0 (134.0–230.2)
Estimated blood loss (mL), median (IQR)	87.0 (59.8–119.0)
Fascial defect (cm), mean ± SD	3.6 ± 0.7
Subcutaneous fat thickness (mm), median (IQR)	17.5 (12.8–22.4)
Length of hospital stay (days), median (IQR)	6.0 (4.0–7.0)
Sex, *n* (%)	Male 266 (61.6%); Female 166 (38.4%)
Smoking, *n* (%)	Yes 107 (24.8%); No 325 (75.2%)
Diabetes, *n* (%)	Yes 104 (24.1%); No 328 (75.9%)
ASA class, *n* (%)	I: 72 (16.7%); II: 211 (48.8%); III: 107 (24.8%); IV: 42 (9.7%)
Nutritional status, *n* (%)	Good 288 (66.7%); Moderate risk 132 (30.6%); Malnutrition 12 (2.8%)
Stoma type, *n* (%)	Loop 399 (92.4%); End 33 (7.6%)
Initial stoma indication, *n* (%)	Rectal cancer 143 (33.1%); Colon cancer 92 (21.3%); Diverticulitis 56 (13.0%); Anastomotic leak protection 49 (11.3%); IBD 36 (8.3%); Trauma 32 (7.4%); Other 24 (5.6%)
Neoadjuvant therapy, *n* (%)	Yes 71 (16.4%); No 361 (83.6%)
Adjuvant oncologic therapy, *n* (%)	Yes 160 (37.0%); No 272 (63.0%)
Mechanical bowel preparation, *n* (%)	Performed 259 (59.9%); Not performed 173 (40.1%)
Oral antibiotic preparation, *n* (%)	Performed 170 (39.4%); Not performed 262 (60.6%)
Prophylactic IV antibiotic, *n* (%)	Given 398 (92.1%); Not given 34 (7.9%)
Surgical approach, *n* (%)	Open 331 (76.6%); Laparoscopic/robotic/conversion 101 (23.4%)
Wound closure method, *n* (%)	Purse-string 202 (46.8%); Primary closure 203 (47.0%); Left open 27 (6.3%)
Anastomotic technique, *n* (%)	Stapled/circular 281 (65.0%); Hand-sewn 135 (31.3%); Combined 16 (3.7%)
Closed suction drain, *n* (%)	Placed 152 (35.2%); Not placed 280 (64.8%)
Perfusion assessment method, *n* (%)	Clinical judgment alone 367 (85.0%); ICG angiography 53 (12.3%); Doppler ultrasound 12 (2.8%)

**Table 2:** Univariate comparison of patients with (*n* = 35) and without (*n* = 397) SSI.

Variable	SSI(+) *n* = 35	SSI(−) *n* = 397	SSI Incidence (Row %)	*p*	Test
Age (years), median (IQR)	63.0 (51.5–70.5)	59.0 (51.0–68.0)	—	0.458	MWU
BMI (kg/m^2^), mean ± SD	28.6 ± 4.5	27.4 ± 3.7	—	0.088	*t*-test
Female sex, *n* (%)	18 (51.4%)	148 (37.3%)	18/166 = 10.8%	0.142	χ^2^(Y)
ASA III–IV, *n* (%)	14 (40.0%)	135 (34.0%)	14/149 = 9.4%	0.596	χ^2^(Y)
Diabetes present, *n* (%)	13 (37.1%)	91 (22.9%)	13/104 = 12.5%	0.093	χ^2^(Y)
Malnutrition/moderate risk, *n* (%)	14 (40.0%)	130 (32.7%)	14/144 (9.7%)	0.493	χ^2^(Y)
Adjuvant oncologic therapy, *n* (%)	20 (57.1%)	140 (35.3%)	20/160 = 12.5%	0.017	χ^2^(Y)
Neoadjuvant therapy, *n* (%)	6 (17.1%)	65 (16.4%)	6/71 = 8.5%	1.000	χ^2^(Y)
MBP performed, *n* (%)	21 (60.0%)	238 (59.9%)	21/259 = 8.1%	1.000	χ^2^(Y)
Oral antibiotic prep, *n* (%)	5 (14.3%)	165 (41.6%)	5/170 = 2.9%	0.003	χ^2^(Y)
Prophylactic IV antibiotic, *n* (%)	32 (91.4%)	366 (92.2%)	32/398 = 8.0%	0.748 †	Fisher
Loop ileostomy (ref: end), *n* (%)	34 (97.1%)	365 (91.9%)	34/399 = 8.5%	0.502 †	Fisher
Smoking, *n* (%)	10 (28.6%)	97 (24.4%)	10/107 (9.3%)	0.734	χ^2^(Y)
Open surgical approach, *n* (%)	29 (82.9%)	302 (76.1%)	29/331 = 8.8%	0.483	χ^2^(Y)
Closed suction drain used, *n* (%)	19 (54.3%)	133 (33.5%)	19/152 = 12.5%	0.021	χ^2^(Y)
Purse-string closure, *n* (%)	17 (48.6%)	185 (46.6%)	17/202 = 8.4%	0.784 ‡	FFH
Primary (linear) closure, *n* (%)	15 (42.9%)	188 (47.4%)	15/203 = 7.4%		
Left open, *n* (%)	3 (8.6%)	24 (6.0%)	3/27 = 11.1%		
Operative duration (min), median (IQR)	71 (58–80)	79 (65–91)	—	0.023	MWU
IBD (initial stoma indication), *n* (%)	2 (5.7%)	34 (8.6%)	2/36 (5.6%)	0.756	Fisher

IQR: interquartile range; MWU: Mann–Whitney U; χ^2^(Y): Yates-corrected chi-square (2 × 2, all expected cells ≥ 5); FFH: Fisher–Freeman–Halton exact test. ‡ Fisher–Freeman–Halton exact test for the three-category wound-closure comparison. † Fisher’s exact test used because an expected cell count was <5. Row percentages give the SSI incidence within each exposure category.

## Data Availability

The dataset analyzed in this study contains potentially identifying clinical information from a single-center retrospective cohort and is therefore not publicly available. De-identified data supporting the findings of this study are available from the corresponding author upon reasonable request and with permission of the Sancaktepe Şehit Prof. Dr. İlhan Varank Training and Research Hospital Scientific Research Ethics Committee. The complete statistical analysis code used to regenerate all reported tables (Table 1, Table 2, Table 3, Table 4 and Table 5) and figures (Figure 3 and Figure 4) directly from the locked, de-identified patient-level analysis dataset (*n* = 432) is provided as Supplementary File S1, together with the full multivariable model output, the STROBE checklist, and ROC coordinate data in the accompanying Supplementary Material.

## Supplementary Materials

The following supporting information can be downloaded at: https://www.mdpi.com/article/10.3390/jcm15197436/s1, Table S1: STROBE Statement—checklist of items that should be included in reports of cohort studies; Table S2: Full multivariable model output; Table S3: ROC coordinate data—corrected primary model; Table S4: Baseline characteristics by MBP status and by oral antibiotic preparation status; Initial stoma indication, by MBP status and by oral antibiotic preparation status Table S5: Organism distribution among SSI cases, by oral antibiotic preparation status; Table S6. Full set of intraoperative variables evaluated for association with SSI; Supplementary File S1: Statistical analysis code.
