# Supplementary material for: Oral Antibiotic Preparation Is Associated with Lower Surgical Site Infection After Ileostomy Closure: A Retrospective Cohort Study
Source: J Clin Med. 2026 Sep 24;15(19):7436. doi: 10.3390/jcm15197436 (PMC13665892; doi:10.3390/jcm15197436)
Supplement: Supplementary file 1 [file jcm-15-07436-s001.zip › jcm-4521443-supplementary.pdf]

Supplementary Materials (Revised)

Oral Antibiotic Preparation Is Associated With Lower Surgical Site Infection After Ileostomy Closure

This replaces the previous supplementary file, which inadvertently contained only duplicates of the main-text tables (see Response to Reviewer 1, Major Comment 14).

Table S1. STROBE Statement — checklist of items that should be included in reports of cohort studies

Section	Item No.	Recommendation	Reported on
Title and abstract	1(a)	Indicate the study's design with a commonly used term in the title or the abstract	p. 1, lines 2–3 (title); p. 1, line 9
	1(b)	Provide an informative and balanced summary of what was done and found	p. 1, lines 4–27
Introduction – Background	2	Explain the scientific background and rationale for the investigation being reported	p. 1, lines 29–45
Introduction – Objectives	3	State specific objectives, including any prespecified hypotheses	p. 1, lines 46–49
Methods – Study design	4	Present key elements of study design early in the paper	p. 2, lines 50–52
Methods – Setting	5	Describe setting, locations, and relevant dates, including periods of recruitment, exposure, follow-up, and data collection	p. 2, lines 52–58
Methods – Participants	6(a)	Give eligibility criteria, sources and methods of selection of participants	p. 2, lines 61–70; Figure 1, p. 3, line 83
Methods – Participants	6(b)	For matched studies, give matching criteria and number of exposed/unexposed	N/A (not a matched design)
Methods – Variables	7	Clearly define all outcomes, exposures, predictors, confounders, effect modifiers	p. 3, lines 84–109 (2.2, 2.3)
Methods – Data sources/measurement	8	For each variable of interest, give sources of data and details of methods of assessment	p. 2, lines 71–77 (2.1); p. 3, lines 103–109 (2.3)
Methods – Bias	9	Describe any efforts to address potential sources of bias	p. 2, lines 76–81 (2.1); p. 11, lines 299–328 (Limitations)
Methods – Study size	10	Explain how the study size was arrived at	p. 2, lines 52–53 (2.1); p. 1, lines 17–19 and p. 10, lines 255–257 (MBP effect interpreted via its 95% CI rather than a post hoc power calculation)
Methods – Quantitative variables	11	Explain how quantitative variables were handled; describe groupings	p. 4–5, lines 127–158 (2.5); Table 3 footnote, p. 8, lines 209–211
Methods – Statistical methods	12(a)	Describe all statistical methods, including those used to control for confounding	p. 4–5, lines 127–147 (2.5)
	12(b)	Describe any methods used to examine subgroups and interactions	p. 10, lines 247–252 (Table 5; interaction test)
	12(c)	Explain how missing data were addressed	p. 2, lines 69–70 (2.1)

Section	Item No.	Recommendation	Reported on
	12(d)	If applicable, describe analytical methods taking account of sampling strategy	N/A (consecutive cohort)
	12(e)	Describe any sensitivity analyses	p. 4, lines 137–147 (2.5)
Results – Participants	13(a)	Report numbers of individuals at each stage of study	Figure 1, p. 3, line 83
	13(b)	Give reasons for non-participation at each stage	Figure 1, p. 3, line 83; p. 2, lines 61–68 (2.1)
	13(c)	Consider use of a flow diagram	Figure 1, p. 3, line 83
Results – Descriptive data	14(a)	Give characteristics of study participants and information on exposures/confounders	Table 1, p. 5, line 163
	14(b)	Indicate number of participants with missing data for each variable	p. 2, lines 69–70 (2.1)
Results – Descriptive data	14(c)	Summarise follow-up time (e.g., average and total amount)	p. 2, lines 57–58 (2.1)
Results – Outcome data	15	Report numbers of outcome events or summary measures	p. 6, line 164; Table 2, p. 6, line 176; Table 4, p. 9, line 241
Results – Main results	16(a)	Give unadjusted and adjusted estimates and their precision; state confounders adjusted for	Table 2, p. 6, line 176; Table 3, p. 7, line 201; text p. 6–7, lines 180–183
	16(b)	Report category boundaries when continuous variables were categorized	p. 3, lines 91–109 (2.3); Table 1, p. 5, line 163
	16(c)	Translate relative risk into absolute risk for a meaningful time period, if applicable	p. 1, lines 14–15 (Abstract); p. 6, lines 170–172 (Results)
Results – Other analyses	17	Report other analyses done (subgroups, interactions, sensitivity analyses)	Table 3, p. 7–8, lines 201–211; Table 5, p. 10, lines 247–252
Discussion – Key results	18	Summarise key results with reference to study objectives	p. 10, lines 254–264 (Discussion, para. 1)
Discussion – Limitations	19	Discuss limitations, sources of bias/imprecision, direction/magnitude of bias	p. 11, lines 299–328 (Limitations)
Discussion – Interpretation	20	Give cautious overall interpretation considering objectives, limitations, multiplicity, similar studies	p. 10–11, lines 254–298 (Discussion); p. 11, lines 330–335 (Conclusions)
Discussion – Generalisability	21	Discuss generalisability of the study results	p. 11, lines 325–328 (Limitations, final sentences)
Other information – Funding	22	Give source of funding and role of funders	p. 12, lines 346–347 (Funding statement)

Page and line numbers refer to the manuscript file as submitted for this revision, with continuous line numbering enabled; section, table, and figure references are also given so that each item remains locatable if pagination shifts at typesetting.

Table S2. Full multivariable model output

S2a. Primary model — variables entered and removed

No variables were removed from the primary model; MBP and oral antibiotic preparation were forced in as pre-specified exposures, and adjuvant oncologic therapy and closed suction drain use were entered as the covariates significant on the corrected univariate analysis (Table 2). No candidate covariate was dropped during fitting.

Term	Coef.	SE	z	p	OR	95% CI
Intercept	-2.900	0.330	-8.79	<0.001	—	—
MBP	0.191	0.376	0.51	0.610	1.21	0.58–2.53
Oral antibiotic prep	-1.561	0.496	-3.14	0.002	0.21	0.08–0.56
Adjuvant oncologic therapy	0.928	0.368	2.52	0.011	2.53	1.23–5.20
Closed suction drain	0.846	0.365	2.32	0.021	2.33	1.14–4.77

All coefficients, standard errors, z statistics, P-values, odds ratios, confidence intervals, and model-performance measures were obtained directly from the fitted statsmodels model objects.

S2b. Sensitivity (backward-elimination) model — elimination sequence

Step	Variable considered for removal	p (Wald)	Action
0	Full candidate set: oral antibiotic prep, adjuvant oncologic therapy, closed suction drain	—	Starting model (all univariate p<0.05 covariates, MBP not forced in)
1	(all three variables p<0.05)	Oral abx 0.002; Adjuvant 0.012; Drain 0.019	No removal — final model

Operative duration was evaluated in a separate sensitivity model by adding it to the primary multivariable model; see Section 2.5 of the manuscript and Supplementary Table S2e.

S2c. Diabetes/ASA-adjusted model (full statsmodels output)

Term	Coef.	SE	z	p	OR	95% CI
Intercept	-2.327	0.323	-7.19	<0.001	—	—
MBP	0.191	0.369	0.52	0.604	1.21	0.59–2.49
Oral antibiotic prep	-1.497	0.499	-3.00	0.003	0.22	0.08–0.60
Diabetes	0.863	0.497	1.74	0.083	2.37	0.90–6.28
ASA III–IV	-0.209	0.487	-0.43	0.667	0.81	0.31–2.10

Model fit: Pseudo R^2 (McFadden) = 0.063; AUC = 0.694; n = 432.

S2d. Firth penalized-likelihood logistic regression — primary model (full output)

Term	OR	95% CI	p
Intercept	0.057	0.027–0.122	<0.001
MBP	1.195	0.582–2.453	0.627
Oral antibiotic prep	0.227	0.089–0.582	0.002
Adjuvant oncologic therapy	2.485	1.232–5.011	0.011
Closed suction drain	2.293	1.141–4.609	0.020

Fit by modified-score iteratively reweighted least squares (Firth, 1993); converged in 8 iterations. Implemented directly (no packaged Firth-logistic library was available in the analysis environment); the complete analysis code is provided as Supplementary File S7.

S2e. Operative-duration-adjusted model (full statsmodels output)

Term	Coef.	SE	z	p	OR	95% CI
Intercept	-2.850	0.460	-6.196	<0.001	—	—
MBP	0.201	0.380	0.530	0.595	1.223	0.58–2.57
Oral antibiotic prep	-1.523	0.509	-2.992	0.003	0.218	0.080–0.588
Operative duration (per minute)	-0.024	0.010	-2.444	0.014	0.976	0.957–0.995

Term	Coef.	SE	z	p	OR	95% CI
Adjuvant oncologic therapy	0.993	0.374	2.658	0.008	2.698	1.30–5.62
Closed suction drain	0.951	0.375	2.535	0.011	2.587	1.24–5.39

All coefficients, standard errors, z statistics, P-values, odds ratios, confidence intervals, and model-performance measures were obtained directly from the fitted statsmodels model objects. Model fit: Pseudo R^2 (McFadden) = 0.106; AUC = 0.751; n = 432.

S2f. IBD-adjusted model (full statsmodels output)

Term	Coef.	SE	z	p	OR	95% CI
Intercept	-2.350	0.330	-7.12	<0.001	—	—
MBP	0.196	0.388	0.506	0.613	1.217	0.57–2.61
Oral antibiotic prep	-1.570	0.507	-3.099	0.002	0.208	0.077–0.561
IBD (initial stoma indication)	-0.072	0.793	-0.090	0.928	0.931	0.197–4.406

All coefficients, standard errors, z statistics, P-values, odds ratios, confidence intervals, and model-performance measures were obtained directly from the fitted statsmodels model objects. Model fit: Pseudo R^2 (McFadden) = 0.052; AUC = 0.712; n = 432.

Table S3. ROC coordinate data — corrected primary model

Threshold	1 − Specificity (FPR)	Sensitivity (TPR)
≥0.276	0.033	0.143
≥0.240	0.068	0.229
≥0.141	0.129	0.371
≥0.131	0.202	0.543
≥0.119	0.265	0.714
≥0.111	0.315	0.771
≥0.062	0.385	0.800
≥0.061	0.529	0.857
≥0.051	0.655	0.886
≥0.033	0.720	0.886
≥0.030	0.773	0.914
≥0.027	0.799	0.914
≥0.025	0.819	0.943
≥0.011	1.000	1.000

AUC = 0.745 (corrected primary model: MBP, oral antibiotic preparation, adjuvant oncologic therapy, closed suction drain).

Table S4a. Baseline characteristics by MBP status and by oral antibiotic preparation status

By MBP status

Variable	MBP performed (n=259)	MBP not performed (n=173)	p
Age, median (IQR)	61.0 (51.5–70.0)	57.0 (49.0–67.0)	0.111
BMI, mean ± SD	27.5 ± 3.6	27.5 ± 3.9	0.945
Female sex, n (%)	112 (43.2%)	54 (31.2%)	0.016

Variable	MBP performed (n=259)	MBP not performed (n=173)	p
ASA III–IV, n (%)	92/259 (35.5%)	57/173 (32.9%)	0.654
Diabetes, n (%)	63 (24.3%)	41 (23.7%)	0.973
Poor nutritional status, n (%)	86 (33.2%)	58 (33.5%)	0.218*
Smoking, n (%)	60 (23.2%)	47 (27.2%)	0.406
End ileostomy, n (%)	23 (8.9%)	10 (5.8%)	0.316
Open surgical approach, n (%)	197 (76.1%)	134 (77.5%)	0.525*
Ostomy duration, median (IQR)	175.0 (136.0–232.0)	171.0 (128.0–228.0)	0.298
Initial stoma indication, n (%) — see Table S4b below			0.146*
Wound closure technique (purse-string/primary/open), n (%)	115 (44.4%) / 125 (48.3%) / 19 (7.3%)	87 (50.3%) / 78 (45.1%) / 8 (4.6%)	0.331*
Year of surgery (2022–2025), n (%)	53/66/74/66	49/40/43/41	0.307*

By oral antibiotic preparation status

Variable	Oral abx given (n=170)	Oral abx not given (n=262)	p
Age, median (IQR)	61.0 (52.0–68.0)	58.0 (50.0–69.0)	0.337
BMI, mean ± SD	27.9 ± 3.3	27.2 ± 4.0	0.057
Female sex, n (%)	64 (37.6%)	102 (38.9%)	0.867
ASA III–IV, n (%)	62 (36.5%)	87/262 (33.2%)	0.553
Diabetes, n (%)	42 (24.7%)	62 (23.7%)	0.895
Poor nutritional status, n (%)	56 (32.9%)	88 (33.6%)	0.970*
Smoking, n (%)	42 (24.7%)	65 (24.8%)	1.000
End ileostomy, n (%)	17 (10.0%)	16 (6.1%)	0.193
Open surgical approach, n (%)	138 (81.2%)	193 (73.7%)	0.301*
Ostomy duration, median (IQR)	177.5 (134.5–235.5)	171.5 (134.0–227.5)	0.700
Initial stoma indication, n (%) — see Table S4b below			0.840*
Wound closure technique (purse-string/primary/open), n (%)	76 (44.7%) / 86 (50.6%) / 8 (4.7%)	126 (48.1%) / 117 (44.7%) / 19 (7.3%)	0.351*
Year of surgery (2022–2025), n (%)	39/42/46/43	63/64/71/64	0.993*

**Multi-category chi-square across all levels (ASA I–IV, nutritional status good/moderate/malnutrition, surgical approach open/laparoscopic/robotic/conversion, stoma indication 7-cat, closure technique 3-cat, year 4-cat), not a 2×2 comparison. Groups are reasonably balanced overall; women were more frequent in the MBP-performed group (p=0.016), which is discussed as a possible residual confounder in the Limitations. The ASA III–IV percentages by MBP and oral antibiotic status shown above have been corrected; the corresponding two-category comparisons (Yates-corrected χ^2) are P = 0.654 (by MBP status) and P = 0.553 (by oral antibiotic status), neither reaching significance.*

Table S4b. Initial stoma indication, by MBP status and by oral antibiotic preparation status

Indication	MBP+ (n=259)	MBP− (n=173)	Oral+ (n=170)	Oral− (n=262)
Rectal cancer	82 (31.7%)	61 (35.3%)	55 (32.4%)	88 (33.6%)
Colon cancer	53 (20.5%)	39 (22.5%)	37 (21.8%)	55 (21.0%)

Indication	MBP+ (n=259)	MBP− (n=173)	Oral+ (n=170)	Oral− (n=262)
Diverticulitis	37 (14.3%)	19 (11.0%)	19 (11.2%)	37 (14.1%)
Anastomotic-leak protection	29 (11.2%)	20 (11.6%)	23 (13.5%)	26 (9.9%)
IBD	29 (11.2%)	7 (4.0%)	16 (9.4%)	20 (7.6%)
Trauma	16 (6.2%)	16 (9.2%)	12 (7.1%)	20 (7.6%)
Other	13 (5.0%)	11 (6.4%)	8 (4.7%)	16 (6.1%)

χ^2 across all 7 categories: p=0.146 (MBP+ vs. MBP−), p=0.840 (Oral+ vs. Oral−) — see Table S4a. Distributions are closely similar between groups and did not drive either p-value.

Table S5. Organism distribution among SSI cases, by oral antibiotic preparation status

Organism / result	Oral antibiotic given (n=5 SSI)	Oral antibiotic not given (n=30 SSI)
Enterococcus faecalis	2	5
Escherichia coli	1	5
Staphylococcus aureus	1	2
Klebsiella pneumoniae	0	2
Polymicrobial growth	0	2
No growth (culture negative)	0	5
Culture not obtained	1	9

Descriptive only; the exposed arm contains only 5 SSI events, too few for a formal statistical comparison of organism distribution.

Table S6. Full set of intraoperative variables evaluated for association with SSI

Variable	p	Test
Bowel segment opened (ileum/cecum/colon)	0.338	χ^2 (3-cat)
Bowel perfusion adequacy (good/questionable/inadequate)	0.659	χ^2 (3-cat)
Perfusion assessment method (clinical/ICG/Doppler)	0.654	χ^2 (3-cat)
Surgical approach (open/lap/robotic/conversion, 4-cat)	0.142	χ^2 (4-cat)
Anastomotic technique (stapled/hand-sewn/combined)	0.066	χ^2 (3-cat)
Wound closure method (3-cat)	0.784	FFH
Closed suction drain use	0.021	χ^2 (Yates)
Fascial defect size (cm)	0.900	t-test
Subcutaneous fat thickness (mm)	0.688	MWU
Estimated blood loss (mL)	0.451	MWU
Operative duration (min)	0.023	MWU
Intraoperative complication (none/serosal injury/enterotomy/bleeding)	0.387	χ^2 (4-cat)

Consistent with Section 2.3/2.4 of the revised manuscript, none of these except closed suction drain use and operative duration reached p<0.05; drain use was retained as a covariate (Table 3); operative duration was not entered into the primary model but was instead evaluated in a separate operative-duration-adjusted sensitivity model (see Response to Reviewer 1, Major Comment 3).

Supplementary File S1. Statistical analysis code

The complete statistical analysis code (Python) used to generate the results in this manuscript is provided as a separate file, Supplementary_Code_S7.py. The script covers, in order: data loading and cleaning; Table 1 (demographic and clinical characteristics of the entire cohort); the univariate tests in Table 2 (χ^2 with Yates correction, Fisher's exact, Fisher–Freeman–Halton, Mann-Whitney U, and t-tests as appropriate to each variable); the primary, backward-elimination sensitivity, diabetes/ASA-adjusted, operative-duration-adjusted, and IBD-adjusted logistic regression models; the Firth penalized-likelihood model; the MBP × oral antibiotic preparation interaction model; Wilson score confidence intervals for the strata in Table 5; and the ROC/AUC and covariate-pattern-based calibration and goodness-of-fit calculations underlying Figures 3 and 4 and the Hosmer–Lemeshow analogue reported in Table 3; and Table 4 (clinical course and microbiological profile of the SSI cases, with denominators stated explicitly for culture-based rows). Table 1 and Table 4 are generated by the same script and the same locked dataset as Tables 2, 3 and 5 and Figures 3 and 4; no value in any table was entered or edited by hand. The script does not contain patient identifiers or any other personally identifiable information; it expects a de-identified analysis dataset as input and is provided for methodological transparency and reproducibility.
